# Genomic characterization of prophage elements in Clostridium clostridioforme: an understudied component of the intestinal microbiome

**DOI:** 10.1099/mic.0.001486

**Published:** 2024-08-12

**Authors:** Suzanne Humphrey, Angeliki Marouli, Katja Thümmler, Margaret Mullin, Leighton Pritchard, Daniel M. Wall

**Affiliations:** 1 Strathclyde Institute of Pharmacy and Biomedical Sciences, University of Strathclyde, Glasgow, G4 0RW, UK; 2 School of Infection and Immunity, College of Medical and Veterinary Sciences, University of Glasgow, Glasgow, G12 8TA, UK; 3 CAF Electron Microscopy Unit (MVLS College Research Facilities), University of Glasgow, Glasgow, G12 8QQ, UK

**Keywords:** bacteriophage, *C. clostridioforme*, dysbiosis, *Enterocloster clostridioformis*, microbiome, TEM

## Abstract

Genome sequencing of *Clostridium clostridioforme* strain LM41 revealed the presence of an atypically high proportion of mobile genetic elements for this species, with a particularly high abundance of prophages. Bioinformatic analysis of prophage sequences sought to characterize these elements and identify prophage-linked genes contributing to enhanced fitness of the host bacteria in the dysbiotic gut. Using PHASTER, PhageScope and manual curation, this work has identified 15 prophages: 4 predicted to be intact, 2 predicted to be defective and 9 which are unclassified. Quantitative PCR (qPCR) analysis revealed spontaneous release of four of the LM41 prophages (φ1, φ2, φ4 and φ10) into the culture supernatant, with virion-like particles visualized using transmission electron microscopy. The majority (12/14) of these particles had morphology akin to podoviruses, which is consistent with morphology predictions for φ1 and φ4. We observed diversity in the lysogeny mechanisms utilized by the prophages, with examples of the classical λ-like CI/Cro system, the ICE*Bs*1 ImmR/ImmA-like system and the Mu-like C/Ner system. Classical morons, such as toxins or immune evasion factors, were not observed. We did, however, identify a variety of genes with roles in mediating restriction modification and genetic diversity, as well as some candidate genes with potential roles in host adaptation. Despite being the most abundant entities in the intestine, there is a dearth of information about phages associated with members of the microbiome. This work begins to shed light on the contribution of these elements to the lifestyle of *C. clostridioforme* LM41.

## Data Summary

Two supplementary figures and one supplementary file containing raw data and analaysis outputs are available at 10.6084/m9.figshare.26273704 [1]. The genome sequence for the C. clostridioforme strain LM41 chromosome is available in the National Center for Biotechnology Information (NCBI) GenBank database under accession number CP125626.1.

## Introduction

The intestinal microbiome, consisting of bacteria, viruses, fungi and protozoa, plays an intimate role in contributing to the health and nutrition of its host. The microbial partners in this commensal relationship aid the host in a variety of ways, including nutrient extraction [2], modulation of the immune and nervous systems via the activity of microbially derived factors [3,5] and by providing a barrier against colonization of the gut with intestinal pathogens [6,7]. Disruption of the diversity and richness in bacterial species comprising the microbiome (known as dysbiosis) can occur through a variety of factors, including host genetics, antibiotic use and diet and lifestyle [8,10].

Several diseases that include a dysbiosis component are associated with signature reductions and blooms of particular bacterial species [11,14]. Currently, the reasons why some species can adapt to and proliferate more readily in response to microbiome perturbation remain unclear. *Clostridium clostridioforme* (recently reclassified as *Enterocloster clostridioformis*) has been noted to proliferate rapidly in the intestines of people with a variety of conditions associated with gut dysbiosis, including obesity, type 2 diabetes and autism spectrum disorder [13, 14,15]. Additionally, Western or high-fat diets, which are established risk factors in obesity and type 2 diabetes development, significantly increase *C. clostridioforme* occurrence in the gut [16], while the species has also been seen to increase post-antibiotic treatment [16,17].

We recently described a novel strain of the gut commensal *C. clostridioforme*, LM41 [18]. In addition to carrying multiple novel secondary metabolite biosynthetic gene clusters, LM41 hosts several novel mobile genetic elements (MGEs), including a 192-kb plasmid, 7 integrative conjugative elements (ICEs), 5 integrative mobilizable elements, 27 IS66 transposases and 29 putative prophages, which could contribute to niche adaptation within the dysbiotic gut environment.

Bacteriophages (phages) are an integral facet of bacterial lifestyles and are widely distributed throughout the human gut microbiota. Estimates of the abundance of viruses and virus-like particles (VLPs) in the intestine range from 10^8^ to 10^10^ VLP/g faeces [19,21], with most being phages [22]. Phages play important roles in regulating bacterial population dynamics, contributing to horizontal gene transfer within and between bacterial species and altering their bacterial hosts’ fitness in the intestinal environment. While virulent phages undergo a strictly lytic replication cycle, which results in phage-mediated bacterial cell death, temperate phages can engage in a secondary lifestyle known as lysogeny. Lysogeny occurs when temperate phages integrate into the bacterial chromosome following infection of the cell, generating a prophage. The prophage remains dormant in the bacterial lysogen, replicating as part of the bacterial chromosome until an environmental signal triggers its induction into the replicative, lytic pathway. Lysogeny was historically considered to be a parasitic relationship on behalf of the phage; however, increasingly studies are demonstrating that temperate phages offer their host bacteria important advantages as trade-offs for the inherent risk associated with their carriage. The integration of a prophage into its bacterial host’s chromosome can permit expression of prophage-encoded factors that alter the host cell phenotype in a process known as lysogenic conversion. These can include virulence factors, such as the Shiga toxin carried by *Escherichia coli* STEC Stx phages, the cholera toxin carried by the *Vibrio cholerae* CTX phage and a variety of staphylococcal toxins carried by *Staphylococcus aureus* phages and phage-inducible chromosomal islands [23,24]. Other well-characterized prophage-encoded factors include the SopE SPI-1 type 3 secretion system effector proteins of *Salmonella* Typhimurium SopEφ and immune system evasion proteins *eib* (serum resistance; λ-like phage) in *E. coli* and *oac* (O-antigen acetylase; Sf6 phage) in *Shigella flexneri* [23]. More recently, prophages have become recognized for the protective effects that their lysogenic lifecycles can have for their host bacterial cell, with some phages encoding factors to modify their host cell’s surface in order to prevent further infection by exogenous phage, e.g. the *gp15*-encoded superinfection exclusion protein of *E. coli* phage HK97 [25], or simply by occupying attachment sites within their lysogen to prevent integration of superinfecting phage. In the latter case, the expression of the CI repressor molecule by the resident phage appears to be sufficient to block the replicative cycle of infecting phages [26], leading to the destruction of the infecting phage when its ability to integrate is impeded by the resident prophage occupying the *att*B site in the bacterial chromosome. Furthermore, at the whole population level, carriage of prophages can be beneficial in enabling lysogenic communities to sample genetic material from other cells owing to the stochastic nature of phage induction [27].

Here, we sought to gain an understanding of the biology of prophages in *C. clostridioforme* LM41. We used a combination of bioinformatic and experimental approaches to reveal the genome structure, functionality and morphology of these prophages. Our findings indicate that *C. clostridioforme* strain LM41 is poly-lysogenic for 15 prophages, the majority of which are predicted to be functional or potentially functional and many of which carry genes with roles in facilitating restriction–modification (RM) and genetic diversity, possibly contributing to the apparent proclivity of LM41 for DNA acquisition. We show via quantitative PCR (qPCR) that 4 of the 15 prophages are spontaneously released from LM41 under standard culture conditions and reveal diversity in particle morphologies using transmission electron microscopy (TEM).

## Methods

### Prophage identification and annotation

For details of *C. clostridioforme* LM41 hybrid Illumina and MinION whole-genome sequencing, refer to Kamat *et al*. [18]. The draft genome has been deposited at GenBank with accession number CP125626.1. Putative prophage sequences present in the LM41 genome were identified using PHASTER [28]. The manual interrogation of 29 PHASTER hits was performed using SnapGene Viewer software (version 5.3, www.snapgene.com) and BLASTp software (https://blast.ncbi.nlm.nih.gov), with hits deemed to be prophages or prophage remnants if they contained gene clusters conforming to one or more of the classical phage genome functional modules (lysis–lysogeny control, DNA replication, packaging and capsid, tail and lysis). Fourteen PHASTER hits were discarded because they either did not correspond to prophages or because multiple hits corresponded to different regions within the same prophage. The resulting 15 regions were annotated using Pharokka [29] (https://usegalaxy.eu/root?tool_id=pharokka) and PhageScope [30] (https://phagescope.deepomics.org). Parameters used for Pharokka annotation were as follows: Pharokka DB v.1.2.0 (downloaded at 2023-08-07 07 : 02 : 08 : 010437); Phanotate gene predictor; E-value threshold for mmseqs PHROGs database, 1E-05. Genome completeness assessments were performed using the integrated CheckV function within PhageScope. Phage regions were categorized based on average amino acid identity (AAI) completeness scores returned via PhageScope: a completeness score of 100 was categorized as ‘functional’; a completeness score of >60–<100 was categorized as ‘unknown’; and a completeness score of <60 was categorized as ‘defective’. The manual inspection of annotated genomes led us to categorize a further five prophages as ‘unknown’ based on unusual features predicted to affect viability (see Results). For the detection of putative promoter sites within lysogeny modules, PhagePromoter [31] (Galaxy server galaxy.bio.di.uminho.pt) was used to search both strands with the following parameters: threshold, 0.5; host bacterial genus, ‘other’; and phage type, ‘temperate’. Phage family (myovirus, siphovirus and podovirus) was selected for each phage as assigned in Table 1. Only hits with scores in the range of 0.87–1.0 were considered. Pharokka and PhageScope genome annotations and PhagePromoter hits are provided in Supplementary Material.

**Table 1.** *C. clostridioforme* LM41 prophage region characteristics

Phage		Location in LM41 genome	Genome size (bp)	DNA strand	Tail features				Closest relative (BLASTn)			
					Baseplate wedge subunit	Tail tape measure	Sheath	Prediction	Description	Identity (%)	Cover (%)	Accession
φ1		194,517–235,362	40 846	Forward	Orf61	nd	nd	Podovirus	*Caudoviricetes* sp. isolate ctRvb1, partial genome	94.43	37	BK050138.1
φ2		301,574–342,165	40 592	Forward	nd	Orf65, Orf66	nd	Siphovirus	*Caudoviricetes* sp. isolate ctdym5, partial genome	95.39	40	BK055266.1
φ3		1,072,300–1,120,599	48 300	Forward	Orf64	Orf59	Orf55	Myovirus	*Enterocloster bolteae* strain CBBP-2	97.5	95	CP053229.1
φ4		1,281,572–1,323,457	41 690	Forward	Orf63	nd	nd	Podovirus	LM41φ1	99.02	74	n/a
φ5		3,073,910–3,122,116	48 207	Reverse	nd	Orf58	nd	Siphovirus	*Blautia pseudococcoides* strain SCSK	79.12	30	CP053228.1
φ6		3,448,526–3,490,199	41 674	Reverse	nd	Orf52	nd	Siphovirus	*Caudoviricetes* sp. isolate cthCz6, partial genome	93.24	38	BK022268.1
φ7		3,885,600–3,901,899	16 300	Reverse	nd	nd	nd	–	*Caudoviricetes* sp. isolate ct1dI13, partial genome	78.11	58	BK021065.1
φ8		3,846,360–3,885,591	39 232	Reverse	Orf47, Or48	nd	Orf39	Myovirus	*Caudoviricetes* sp. isolate ctELP1	79.38	65	BK049563.1
φ9		3,997,064–4,063,629	66 566	Reverse	nd	Orf133	nd	Siphovirus	*Caudoviricetes* sp. isolate ctRyL7, partial genome	91.04	68	BK049246.1
φ10		4,120,662–4,165,801	45 140	Reverse	Orf80, Orf81	Orf75	Orf71	Myovirus	*Caudovirales* sp. isolate ctjI31, partial genome	93.81	71	BK029493.1
φ11		5,193,058–5,226,919	33 862	Reverse	nd	Orf53	nd	Siphovirus	*Caudoviricetes* sp. isolate ctpXm10, partial genome	92.98	48	BK024821.1
φ12		6,190,004–6,237,536	47 533	Forward	nd	Orf63	nd	Siphovirus	*Enterocloster bolteae* strain ATCC BAA-613 chromosome	89.09	39	CP022464.2
φ13		6,632,999–6,769,447	136 449	Forward					n/a	n/a	n/a	n/a
	α	6,632,999–6,684,039	51 041	Forward	nd	Orf65	nd	Siphovirus	*Siphoviridae* sp. isolate ctgM31, partial genome	92.9	75	BK028648.1
	β	6,684,149–6,706,426	22 278	Forward	nd	nd	nd	–	*Caudoviricetes* sp. isolate ct1i×6, partial genome	81.66	26	BK023115.1
	γ	6,706,413–6,714,990	8578	Forward	nd	nd	nd	–	*Lachnoclostridium* sp. YL32 chromosome, complete genome	95.82	96	CP015399.2
	δ	6,715,266–6,749,468	34 203	Forward	Orf174	Orf184, Orf185	Orf180	Myovirus	*Lachnoclostridium* sp. YL32 chromosome, complete genome	97.62	53	CP015399.2
	ε	6,750,116–6,769,447	19 332	Forward	nd	Orf227	nd	Siphovirus	*Siphoviridae* sp. ctquf9, partial genome	86.92	88	BK014815.1
φ14		7,473,893–7,529,677	55 785	Forward	Orf65	Orf61	Orf57	Myovirus	No matches with coverage >1 %	n/a	n/a	n/a
φ15		7,532,147–7,579,680	47 534	Forward	Orf72	Orf68	Orf63	Myovirus	*Lachnoclostridium* sp. YL32 chromosome, complete genome	90.62	46	CP015399.2

ndnot detected

### Bacterial strains and culture conditions

*C. clostridioforme* LM41 was grown in Fastidious Anaerobe Broth (FAB; Neogen) or on FAB agar [FAB supplemented with 1.5 % agar (Formedium)] under anaerobic conditions (10 % H_2_, 10 % CO_2_, 80 % N_2_ and 60–70 % humidity) in an A35 workstation (Don Whitely Scientific) at 37^ ^°C. All media was reduced prior to inoculation. Overnight cultures were first prepared by inoculating 5 ml of pre-reduced FAB with a single colony of LM41 from a freshly streaked plate. Fresh, pre-reduced FAB was subsequently inoculated 1 : 50 (v/v) with the overnight culture and allowed to grow for up to 24 h under anaerobic conditions.

### Induction of *C. clostridioforme* LM41 prophages using DNA-damaging antibiotics

*C. clostridioforme* LM41 was diluted 1 : 50 from an overnight culture into 50 ml FAB and grown to an OD via absorbance at 600 nm (OD_600_) of 0.7 under anaerobic conditions at 37 °C. Cultures were induced by the addition of DNA-damaging antibiotics to a final concentration of 3 µg ml^−1^: mitomycin C (Sigma), norfloxacin (Sigma) or ciprofloxacin (Sigma). An uninduced control culture was also included. All cultures were grown for a further 16–18 h post-induction. Cultures were then centrifuged at 2800 × *g* for 30 min, and the supernatants were filtered through 0.22-µm filters to remove the remaining bacterial cells.

### Extraction and quantification of encapsidated DNA from induced samples

Filtered supernatants were treated with 10 µg ml^−1^ DNase I (Sigma) and 1 µg ml^−1^ RNase A (Sigma) for 30 min at room temperature; then, NaCl was added to a final concentration of 1 M. After incubation for 1 h on ice, the mixture was centrifuged at 11 000 × *g* for 10 min at 4 °C and the supernatant was transferred to a fresh tube. PEG 8000 was added to the supernatant at a final concentration of 10 % (w/v), and the mixture was incubated overnight at 4 °C. Phages were precipitated from the mixture by centrifugation at 11 000 × *g* for 20 min at 4 °C, with the final pellet resuspended in 1 ml phage buffer (1 mM NaCl, 0.05 M Tris pH 7.8, 1 mM MgSO_4_ and 4 mM CaCl_2_). For the extraction of encapsidated DNA, each sample was subject to a further DNase I treatment (20 µg ml^−1^) for 1 h at room temperature to degrade any non-encapsidated DNA in the sample. DNase activity was stopped by the addition of 20 mM ethylenediaminetetraacetic acid (EDTA) (Sigma), with incubation for 10 min at 70 °C. Capsids were then opened by the addition of 50 µg ml^−1^ proteinase K (Sigma) and 1 % SDS to each sample, with incubation at 55 °C for 1 h, mixing at 20-min intervals. The samples were subsequently transferred to fresh microcentrifuge tubes, and an equal volume of phenol-chloroform-isoamyl alcohol (25 : 24 : 1; Sigma) was added to each. Samples were mixed by vortexing followed by centrifugation at 18 000 × *g* for 5 min at 4 °C to allow separation of the phases. The upper phase was transferred to a fresh microcentrifuge tube, and the DNA was precipitated by the addition of 0.1 vol. of 3 M sodium acetate (pH 5.2) and 2.25 vols of ice-cold 100 % ethanol at −80 °C for 16–18 h. Samples were centrifuged at 18 000 × *g* for 20 min at 4 °C, and the pellets were washed once with ice-cold 70 % ethanol before centrifuging once more. After discarding the supernatant, the pellets were air dried prior to resuspension in 50 µl nuclease-free water. Resuspended pellets were stored at 4 °C for 16–18 h to allow sufficient time for solubilization of DNA in each sample; then, the DNA was quantified using a DS-11 spectrophotometer (DeNovix Inc., Wilmington, USA).

### Detection of spontaneous LM41 phage release by qPCR

The presence of phages in the bacterial supernatant was quantified using qPCR. *C. clostridioforme* LM41 was grown for 24 h in FAB under anaerobic conditions at 37 °C. The culture was centrifuged at 2800 × *g* for 30 min and the supernatant filter sterilized through a 0.22 µm-syringe filter to remove the bacterial cells and debris. Equal volumes of supernatant were treated with 10 µg ml^−1^ DNase I (Sigma) in DNase-activating buffer (50 mM Tris-HCl, pH 7.5; 10 mM MgCl_2_) to degrade non-encapsidated DNA, or with an equal volume of DNase-activating buffer (without DNase I) as a control. In parallel, 200 ng of LM41 genomic DNA was digested to confirm enzymatic activity of the DNase enzyme mix (see Fig. S1, available in the online supplementary material). Samples were incubated at 37 °C for 1 h and then heated at 85 °C for 15 min to inactivate the DNase enzyme and lyse any phage capsids present to release encapsidated DNA. Samples were used immediately as template for qPCR analysis.

qPCR was performed using a CFX connect real-time qPCR system (Bio-Rad). Twenty-microlitre reaction mixtures were prepared using the Luna Universal qPCR Master Mix kit (New England Biolabs) as follows: 10 µl 2× Luna qPCR master mix, 7.0 µl nuclease-free H_2_O, 1.0 µl forward primer (final concentration 3 µM), 1.0 µl reverse primer (final concentration 3 µM) and 1.0 µl of either DNase-treated or control supernatant as template. Three technical replicates were performed per sample per primer pair (Table 2). Cycling conditions were 95 °C for 3 min and then 40 cycles of 95 °C (10 s), 60 °C (10 s) and 65 °C (30 s). DNase treatment degrades free bacterial DNA (including cellular prophage copies), leaving only encapsidated DNA. Using the 2^-ΔΔCq^ method, we compared the effect of DNase treatment, normalized using *C. clostridioforme* LM41 small ribosomal protein 10 gene (*s10p*; *rpsJ* equivalent) as the housekeeper. Where phages are present in the supernatant, the difference in Cq values between the treatment groups will be smaller, resulting in higher 2^-ΔΔCq^ values.

**Table 2.** Oligonucleotides used in this study

Primer name	Sequence (5′−3′)	Target	Expected product (bp)
s10p-q-F	AGGATCACAGGTGAGCGGAC	Small ribosomal protein 10 (*rpsJ*)	146
s10p-q-R	GGCTTGGAGCTGTGATGTCG		
LM41phi1-q-F	ACAGCCAGAAAGCGAGCAGA	Prophage 1 *orf1* (integrase)	134
LM41phi1-q-R	TGTCCAGTGATTGCTCCGCA		
LM41phi2-q-F	TATTCTGGCCCTGCTGACGG	Prophage 2 *orf1* (integrase)	129
LM41phi2-q-R	TAGCCCATGACCGCCTCAAG		
LM41phi3-q-F	GGAAGCGGCGAACCTAAAGC	Prophage 3 *orf16* (putative VRR-DNA nuclease)	92
LM41phi3-q-R	TATACAGCCCGTGGAAGCCG		
LM41phi4-q-F	TCGGCCAACTCATTCAATGCT	Prophage 4 *orf3* (putative repressor)	142
LM41phi4-q-R	CGCAAGATGACGAGACAGCAC		
LM41phi5-q-F	AGTCGCTGGATACGCTGGAC	Prophage 5 *orf21* (hypothetical protein)	100
LM41phi5-q-R	TCCGTATCCGTCAAGGTCGC		
LM41phi6-q-F	GATACGGCCAGGGAGCTTGT	Prophage 6 *orf16* (DNA polymerase)	130
LM41phi6-q-R	CGGCGAAGGTGTATCCGTCT		
LM41phi7-q-F	GTATCAAAAGCGGCAGGGGC	Prophage 7 *orf18* (DnaC-like helicase loader)	106
LM41phi7-q-R	TACCGGGATACTCCGCTCCA		
LM41phi8-q-F	CCAGATAGCGGCAAAGCAGC	Prophage 8 *orf23* (putative ParB N-terminal domain-containing protein)	154
LM41phi8-q-R	GTATGCCTCCAGCGGTTCCT		
LM41phi9-q-F	GGAACGCCAACCCGTGGATA	Prophage 9 *orf44* (hypothetical protein)	140
LM41phi9-q-R	GCATGGTTCATCCGCCCAAG		
LM41phi10-q-F	AGCTGCTGCCGAGTTTCTGA	Prophage 10 *orf43* (hypothetical protein/intergenic region)	179
LM41phi10-q-R	GTAAGTGCATACGCGCCACC		
LM41phi11-q-F	ACGCCGGATAAAGGAAGGGG	Prophage 11 *orf17* (HTH DNA-binding protein)	131
LM41phi11-q-R	CCCCGTGATAGGCCATGGTT		
LM41phi12-q-F	GCGGCGGATTCAGAAACTGG	Prophage 12 *orf39* (transcriptional regulator)	89
LM41phi12-q-R	CTATCGTGCCGTCCCGTCTT		
LM41phi13α-q-F	AGGTGTTTGCTTCCCACGGA	Prophage 13 *orf19* (DNA polymerase)	151
LM41phi13α-q-R	AATGCCCCTACTGATCCGCC		
LM41phi13β-q-F	CGCTGTATGGCAAAGGGCAG	Prophage 13 *orf107* (putative type III restriction–modification system subunit)	161
LM41phi13β-q-R	AGACCCCGTTACCAGCATCG		
LM41phi13γ-q-F	ATTAACCCGGCGGATGTGGT	Prophage 13 *orf115* (putative DNA transcription initiation protein)	143
LM41phi13γ-q-R	ATAGGCTCCTGTCGCTGCTG		
LM41phi13δ-q-F	GCATGCTGCATTGTACCGCT	Prophage 13 *orf164* (large terminase)	182
LM41phi13δ-q-R	CGTCCGCTGCTGTCAGGATA		
LM41phi13ε-q-F	CTGACGGCCAGGATAAGGCA	Prophage 13 *orf219* (major capsid protein)	172
LM41phi13ε-q-R	GCCTGAATCAAGCGGCTGTC		
LM41phi14-q-F	AGCTCCAAGCCAAAGCGGTA	Prophage 14 *orf24* (hypothetical protein)	121
LM41phi14-q-R	CCCCGCTGTGTTCTTACGGA		
LM41phi15-q-F	CATGCGGCGGCAGATAACTT	Prophage 15 *orf35* (hypothetical protein)	155
LM41phi15-q-R	TTCCACTGCTCTCTCGCACG		

### Purification of LM41 phages for TEM

Under anaerobic conditions at 37 °C, 1.6 l of *C. clostridioforme* LM41 was grown for 24 h in FAB. The culture was centrifuged at 2800 × *g* for 30 min, and the supernatant was filtered through a 0.22-µm filter to remove the remaining bacterial cells. The supernatant was treated with 10 µg ml^−1^ DNase I for 1 h at room temperature; then, NaCl was added to a final concentration of 1 M. After incubation for 1 h on ice, the mixture was centrifuged at 11 000 × *g* for 10 min at 4°C. PEG 8000 was added to the supernatant at a final concentration of 10 % (w/v), and the mixture was incubated overnight at 4 °C. Phages were precipitated from the mixture by centrifugation at 11 000 × *g* for 20 min at 4 °C, with the final pellet resuspended in 1 ml phage buffer (1 mM NaCl, 0.05 M Tris pH 7.8, 1 mM MgSO_4_ and 4 mM CaCl_2_) and stored at 4 °C.

### TEM

Carbon-filmed 400-mesh copper TEM grids (AGAR Scientific) were glow discharged using a Quorum Q150TES high vacuum coater (20 mA, 30 s). Three microlitres of precipitated phage suspensions was applied to the resulting hydrophilic carbon support films and allowed to adsorb for 3 min. Excess volume was removed by blotting, and the grids were fixed for 5 min in 1 % (w/v) paraformaldehyde in PBS solution. Grids were washed three times with distilled water for 30 s and then stained with 0.5 % (w/v) uranyl formate solution for 30 s. Sample grids were air dried at room temperature and then were examined using the JEOL 1400 FLASH TEM microscope running at 80 kV at the University of Glasgow CAF Electron Microscopy Unit (MVLS College Research Facilities). Digital images were captured at 50–150 K magnification using JEOL TEM Centre software v.1.7.26.3016 and inbuilt 2K × 2K CCD Flash camera. Particle dimension measurements were performed using ImageJ software v.1.54 h (https://imagej.net/ij/).

### Statistical analysis

Statistical analyses were performed as described in the figure legends. All analyses were performed using GraphPad Prism software version 10.1.2. Thresholds were *, P<0.05; **, P<0.01; ***, P<0.001; and *P*>0.05, not significant.

## Results

### *C. clostridioforme* LM41 is a poly-lysogen harbouring 15 prophage regions

**Fig. 1.**
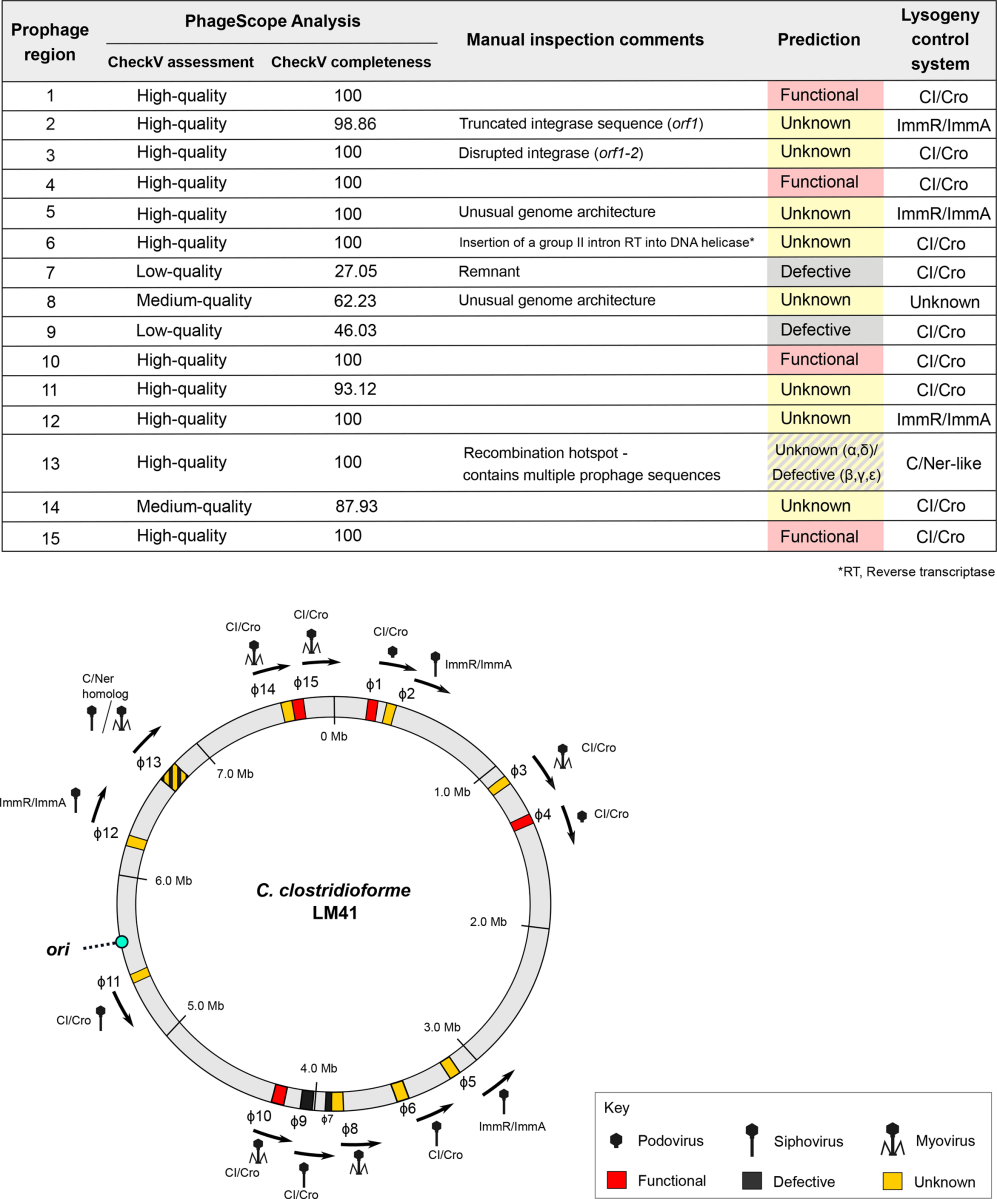
Arrangement of prophages in the *C. clostridioforme* LM41 chromosome. Locations of each of the prophage regions were mapped according to their coordinates in the LM41 chromosome. Coloured areas indicate the presence of prophages predicted to be functional (red), defective (black) or unknown (yellow) using PhageScope genome completeness assessment followed by manual inspection. Region 13 (hatched) is a putative hotspot for phage recombination and is predicted to contain multiple phage sequences, some of which are defective remnants and some of which are functionally unclassified. Arrows indicate the predicted direction of phage packaging. Putative repressor families are indicated for each phage: λ-like CI/Cro systems, ICE*Bs*1 ImmR/ImmA-like systems and phage Mu C/Ner-like systems. Icons represent the predicted particle morphology based on the presence of genes encoding key tail structures: podoviruses, baseplate only; myoviruses, sheath; and siphoviruses, tail tape measure protein but no sheath. The bacterial chromosome origin of replication (*ori*) is indicated by the turquoise circle. Image generated using Inkscape v.1.

We previously reported that PHASTER analysis of the LM41 genome revealed 29 predicted prophage regions [18]. These regions were subsequently interrogated to ascertain the presence of classical prophage-associated functional modules to confirm the hits as phage. Prophage completeness was estimated using AAI comparison via PhageScope [30], followed by manual inspection to ensure that the regions possessed essential functional modules, with genes arranged in the appropriate direction(s) to enable expression as operons. Using these criteria, 15 prophage regions were identified (Figs1  2 and Table 1). The genome organization, size and gene synteny of the majority of these prophages are reminiscent of prophages from other Gram-positive species, in particular the siphoviruses of *S. aureus*. Comparative nucleotide analysis of other *C. clostridioforme* prophage genomes suggests that several prophages, namely, LM41φ1, 4, 5, 7, 8 and 14, are unique to strain LM41 among our reference dataset (see Fig. S2). Four prophages (LM41φ1, φ4, φ10 and φ15) returned completeness scores of 100, suggesting that they are intact and putatively functional. Interestingly, prophages LM41φ1 and LM41φ4 are highly similar to one another (99.02 % ID across 74 % of the phage sequence), with divergence occurring principally in the integrase, *cI/cro* lysogeny region and in the latter half of the replication module.

**Fig. 2.**
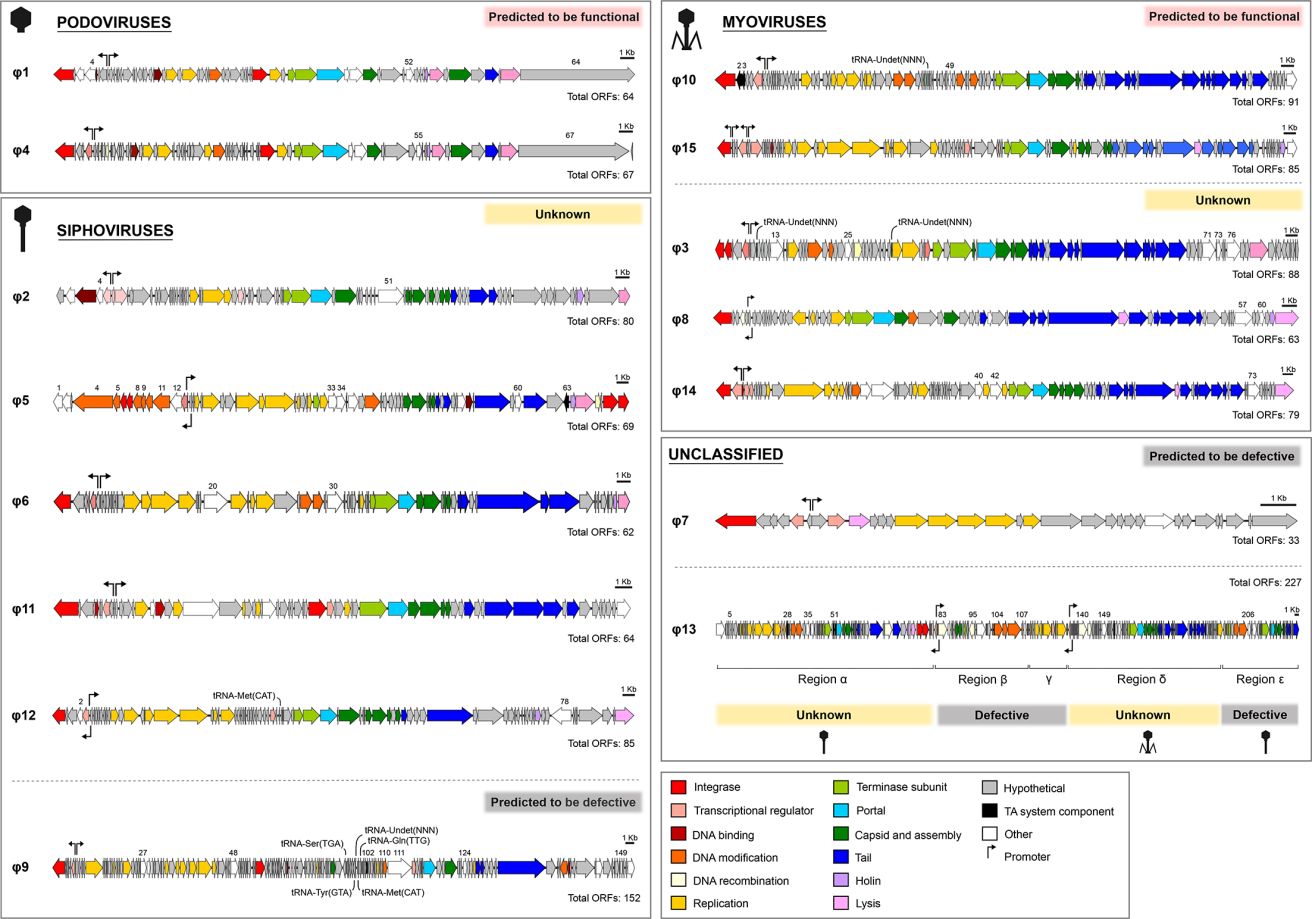
Genome maps of LM41φ1–15. Schematic maps of the ORFs predicted for each of the prophage regions identified in *C. clostridioforme* LM41. Phages are grouped by particle morphology. Genes are coloured according to their predicted functional group with tRNAs indicated. Putative promoters associated with lysogeny control, identified using PhagePromoter, are indicated by black arrows. Numbers denote the presence of potential accessory genes of interest, as described in Table 3. A scale of 1 kb is denoted by the black bars. Images were generated using SnapGene v.6.1.1 and InkScape v.1 software.

Prophage regions LM41φ7 and LM41φ9 returned poor completeness scores (<60), suggesting that these are defective phages due to the loss of parts of their coding sequence. Indeed, LM41φ7 appears to be a prophage remnant, encoding only lysogeny and replication modules before being interrupted by the integrase of LM41φ8.

Regions LM41φ2, φ8, φ11 and φ14 were classified as ‘unknown’ on the basis that they returned completeness scores of >60–<100. In addition, though prophages LM41φ3, φ5, φ6, φ12 and φ13 returned completeness scores of 100, manual inspection of their genomes revealed unusual characteristics predicted to affect prophage viability; hence, we also categorized them as ‘unknown’ pending further investigation.

LM41φ3 exhibits peculiarities in its integrase (*orfs1-2*); namely, that *orf2* carries a putative frameshift mutation that results in a premature stop codon after residue P199, splitting the integrase sequence and likely rendering the prophage defective. Meanwhile, compared to the other prophages, LM41φ5 has an unusual genomic composition, with a larger lysogeny module (*orf1-13*) containing two putative integrase sequences (*orf6-7*) located between proteins comprising a complete type I RM system. In the case of Orf6-7, these putative integrase proteins are smaller than expected for a typical functional integrase (Orf6, 165 aa; Orf7, 167 aa) suggesting that these may be an integrase truncated by mutation. We attempted to investigate this by searching for related sequences to determine whether a premature stop codon had been introduced by single nucleotide polymorphism to φ5; however, a BLASTn search of the sequence encompassing *orf6-7* returned no matches with significant similarity, leaving us unable to resolve this question at this time. φ5 also carries two ORFs predicted to encode recombinases (*orf68-69*) at the terminal end of the prophage sequence. These recombinases are closer in length to that expected for a functional integrase protein (Orf68, 420 aa; Orf69, 282 aa), raising questions as to whether one of these could catalyse the integration of the phage at the *att*B site if indeed the first integrase is non-functional. In addition, we were unable to identify certain components essential for virion packaging in LM41φ5, namely, the large terminase subunit and portal protein, raising questions about the capability of this phage to package its genome into procapsids.

LM41φ6 and LM41φ12 exhibit anomalies in their DNA replication and lysis modules, respectively, on account of insertion of a putative group II intron reverse transcriptase/maturase (LM41φ6, *orf20*; LM41φ12, *orf78*). LM41φ6 *orf20* is positioned between two putative DNA helicases (*orf17* and *orf21*). *orf17* and *orf21* are not duplicated genes as they do not display any significant nucleotide similarity. Rather, BLASTn analysis of *orf17* and *orf21* sequences revealed hits with high similarity to the virulence-associated protein E (putative DNA helicase) from *Caudoviricetes* sp. isolate ctdym5 (Acc: BK055266) (*orf17* : 99.92 % identity, 51 % cover, *E* value 0.0; *orf21* : 99.83 % identity, 47 % cover, *E* value 0.0), suggesting that *orfs17* and *21* are one ORF that has been split by the insertion of the putative group II intron. This likely eliminates the functionality of the helicase protein and renders the phage incapable of initiating replication following activation. In LM41φ12, *orf78* is positioned divergently to its flanking genes, potentially affecting the transcription of the late module as a polycistronic transcript and therefore affecting the ability of the phage to induce host lysis.

Finally, the data obtained for region φ13 were puzzling. A completeness score of 100 was returned for this 136.5-kb region, suggesting potential functionality; however, the manual inspection revealed the presence of a variety of prophage sequences with differing levels of completeness. Some of the sequences within region 13 were reminiscent of Mu-type phage; hence, we divided the 136.5-kb region into sub-regions (α-ε) by matching the predicted functions of the ORFs relative to the functional modules expected for a complete Mu-type phage (i.e. a transposase, ATP-binding or DNA replication protein indicated the likely start of a phage sequence, while a tail or recombinase indicated the likely end). We then refined the regions by searching for hits using BLASTn and assessing completeness using PhageScope (Fig. 2).

Regions φ13β, γ and ε returned low completeness scores (49.4, 18.8 and 41.2, respectively), suggesting that these areas are defective phage remnants. The most complete stretches of prophage genome in this region are the 51.0 kb φ13α (*orf1-79,* completeness score 99.65) and 34.2 kb φ13δ (*orf128–192,* completeness score 94.11) regions (Fig. 2). Prophage φ13α appears to encode most of the required modules for replication and assembly of phage virions. However, despite encoding two putative integrase genes at its 3′ terminus, it lacks a clear lysogeny module and does not encode a Mu transposase C-terminal domain-containing protein to permit transposable replication, suggesting that it may be incomplete. Prophage φ13δ has high similarity to *Clostridium* phage Villandry (BLASTn: 95.82 % identity, 96 % cover, *E* value 0.0, Acc ON453902.1) and is reminiscent of the prototypical transposable phage Mu, encoding putative candidates for a repressor (*orf129*), a *ner*-like transcriptional regulator (*orf139*), a Mu transposase (*orf140*) and a Mor transcription activator family protein (*orf158*) and structural components such as Mu-like prophage I protein (*orf167*), phage Mu protein F-like protein (*orf166*) and Mu-like prophage major head subunit gpT (*orf169*).

### LM41 prophages exhibit diversity in their lysogeny control mechanisms

Our analysis revealed diversity in the lysogeny control mechanisms of the LM41 prophages, with three groups identified: classical λ-like CI/Cro systems [26], ImmR/ImmA-like systems similar to that used by the *Bacillus subtilis* ICE*Bs*1 [32] and systems reminiscent of the CI/Cro-like C/Ner system of *E. coli* phage Mu [33].

Most prophages (10/15) appear to possess lysogeny systems reminiscent of the classical λ-like CI/Cro system. Prophages LM41φ1, φ3, φ4, φ6, φ7, φ9, φ10, φ11, φ14 and φ15 each possess a pair of adjacent divergently transcribed genes in their putative lysogeny modules, several of which are predicted to be helix-turn-helix (HTH) transcriptional regulators, representing probable CI-like repressors. Using PhagePromoter [31], we detected divergent promoters in the intergenic region between these ORFs (Fig. 2), lending support to our hypothesis that these ORFs encode a CI/Cro-like lysogeny switch in these phages.

Prophages LM41φ2, φ5 and φ12 appear to encode systems analogous to the ImmR/ImmA regulatory systems that have been described for *B. subtilis* ICE*Bs*1 [32] and some phage-inducible chromosomal islands in staphylococci [34]. In the lysogeny modules of φ2 and φ12, *orf*4 is predicted to encode an ImmA/IrrE family metallo-endopeptidase which is located directly adjacent to *orf5*. Orf5 is predicted to be a HTH transcriptional regulator, which we propose to be ImmR-like based on its functional prediction and synteny with *immR* from ICE*Bs*1. Importantly, *orfs4–5* are transcribed in the same direction (leftward), while the downstream ORF (*orf6*), predicted to encode a second HTH transcriptional regulator, is transcribed in the rightward direction towards the DNA replication module. Within the intergenic region between *orf5* and *orf6* in φ2, PhagePromoter predicted the presence of two divergent promoters akin to that observed for P*immR* and P*xis* in ICE*Bs*1 (Fig. 2). Conversely, in φ5 and φ12, a pair of convergent promoters were predicted in the intergenic regions between *orfs13–14* (φ5) and *orfs5–6* (φ12), suggesting that transcriptional interference similar to that observed in coliphage 186 [35] may play a role in regulating the lysogenic–lytic control switches in these prophages.

The final lysogeny system that we observed is reminiscent of the C/Ner system of *E. coli* phage Mu. In phage Mu, the 197-aa repressor protein (C) is divergently transcribed from the 76-aa DNA binding protein, Ner, which functions to negatively regulate transcription of the replicative transposition genes [33]. LM41φ13 appears to be a highly plastic region on the LM41 chromosome that contains the multiple Mu-type prophage/remnants arranged consecutively, which we have designated φ13α-ε. In LM41φ13, Orf80, located in region φ13β, is predicted to be a 168-aa HTH transcriptional regulator and is transcribed divergently from the downstream gene, *orf*82, which is also predicted to encode a 51-aa HTH transcriptional regulator (BLASTp: 100 % identity, 98 % cover, *E* value 1e-26, Acc: WP_303009215.1) (Fig. 2). Immediately downstream, *orf*83 is predicted to encode a protein with a Mu transposase C-terminal domain. The similar size, synteny and functional predictions of *orf60*, *orf62* and *orf63* from LM41φ13β with that of the genes encoding C, Ner and the transposase from the classical phage Mu suggest that the functional ancestor of this phage utilized lysogeny regulation mechanism similar to that of phage Mu. A similar arrangement is present in region φ13δ; however, nine ORFs with hypothetical functions are located between the putative *c* (*orf129*) and *ner* (*orf139*) homologues.

### LM41 prophages are predicted to be morphologically diverse

Except the remnants of φ7, φ13β and φ13γ, genome analysis indicated that all LM41 prophages are tailed, with each of the major tail groups represented (Table 1). Prophages φ1 and φ4 are predicted to carry a single tail gene (*orf61* and *orf63*, respectively). Their products are predicted to be baseplate wedge subunits with homology to tail fibre (spike) proteins, suggesting that these may be podoviruses. Tail sheath proteins were observed in the genomes of φ3, φ8, φ10, φ13δ, φ14 and φ15, suggesting that these may be myoviruses with contractile tails. The remaining prophages, φ2, φ5, φ6, φ9, φ11, φ12, φ13α and φ13ε, possessed tape measure proteins but no sheaths, suggesting that they may be siphoviruses.

### Phage particles are spontaneously released from *C. clostridioforme* LM41

**Fig. 3.**
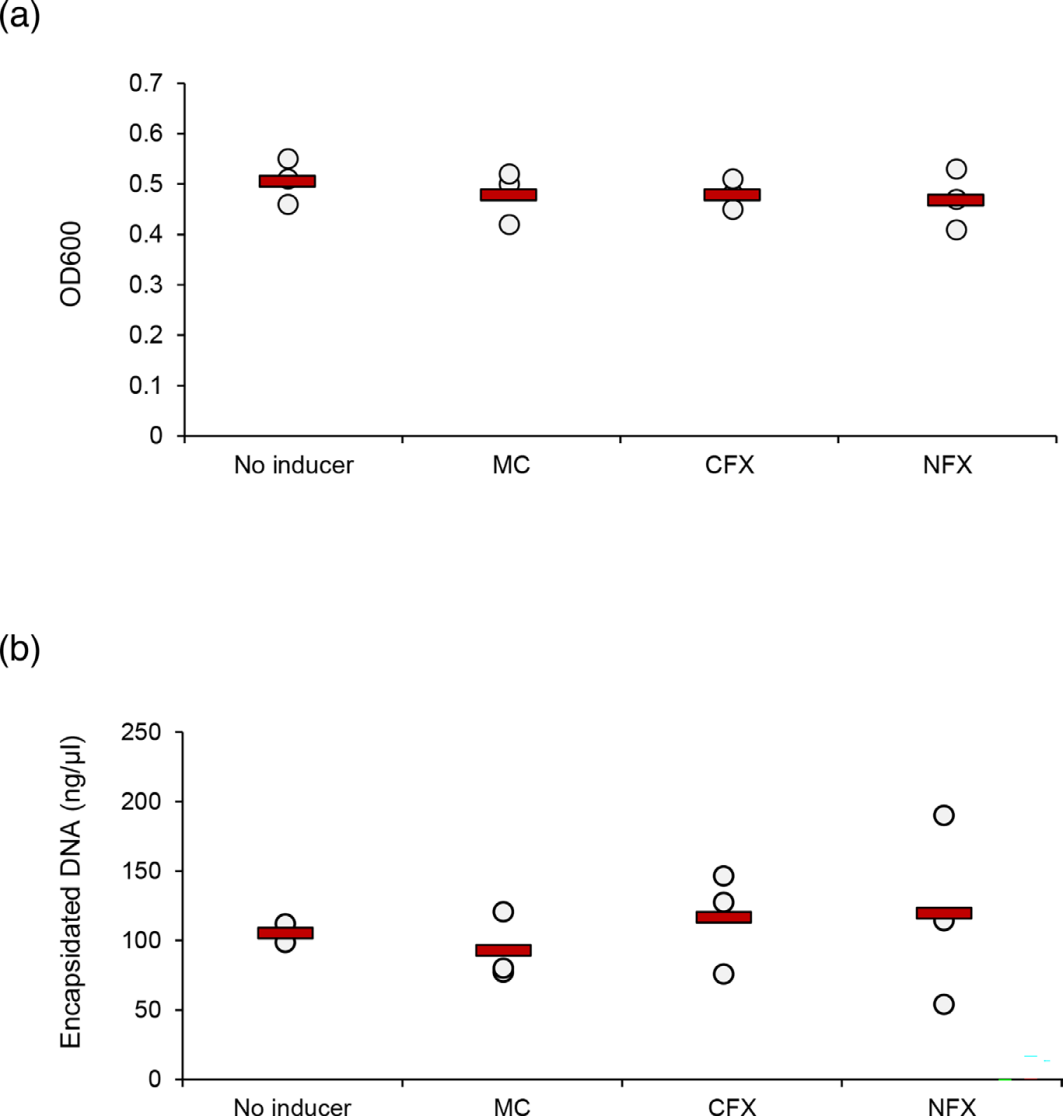
Common SOS response-inducing agents are not potent inducers of *C. clostridioforme* LM41 prophages. *C. clostridioforme* LM41 cultures were induced with common SOS response-inducing chemicals, mitomycin C (MC), ciprofloxacin (CFX) or norfloxacin (NFX), and grown for 16–18 h at 37 °C in anaerobic conditions. An uninduced control sample was also included. OD600 values were obtained for each culture after 16–18 h to determine if phage-induced lysis had occurred (a) and encapsidated DNA was purified and quantified for each culture (b). Data points are from three independent experiments (*n*=3), with mean values shown as red bars. All data were tested for significance using a one-way ANOVA with Tukey post hoc tests; no statistically significant differences were observed between the groups (*P*>0.05).

We sought to determine whether we could identify any of the phages in the supernatant of LM41 cultures. Treatment with classical SOS-response inducers such as mitomycin C, ciprofloxacin and norfloxacin failed to induce lysis of LM41 cultures and did not lead to significantly higher levels of encapsidated phage DNA in induced cultures compared with basal release in untreated cultures (Fig. 3), suggesting that these chemicals are not potent inducers of LM41 prophages under the conditions tested.

**Fig. 4.**
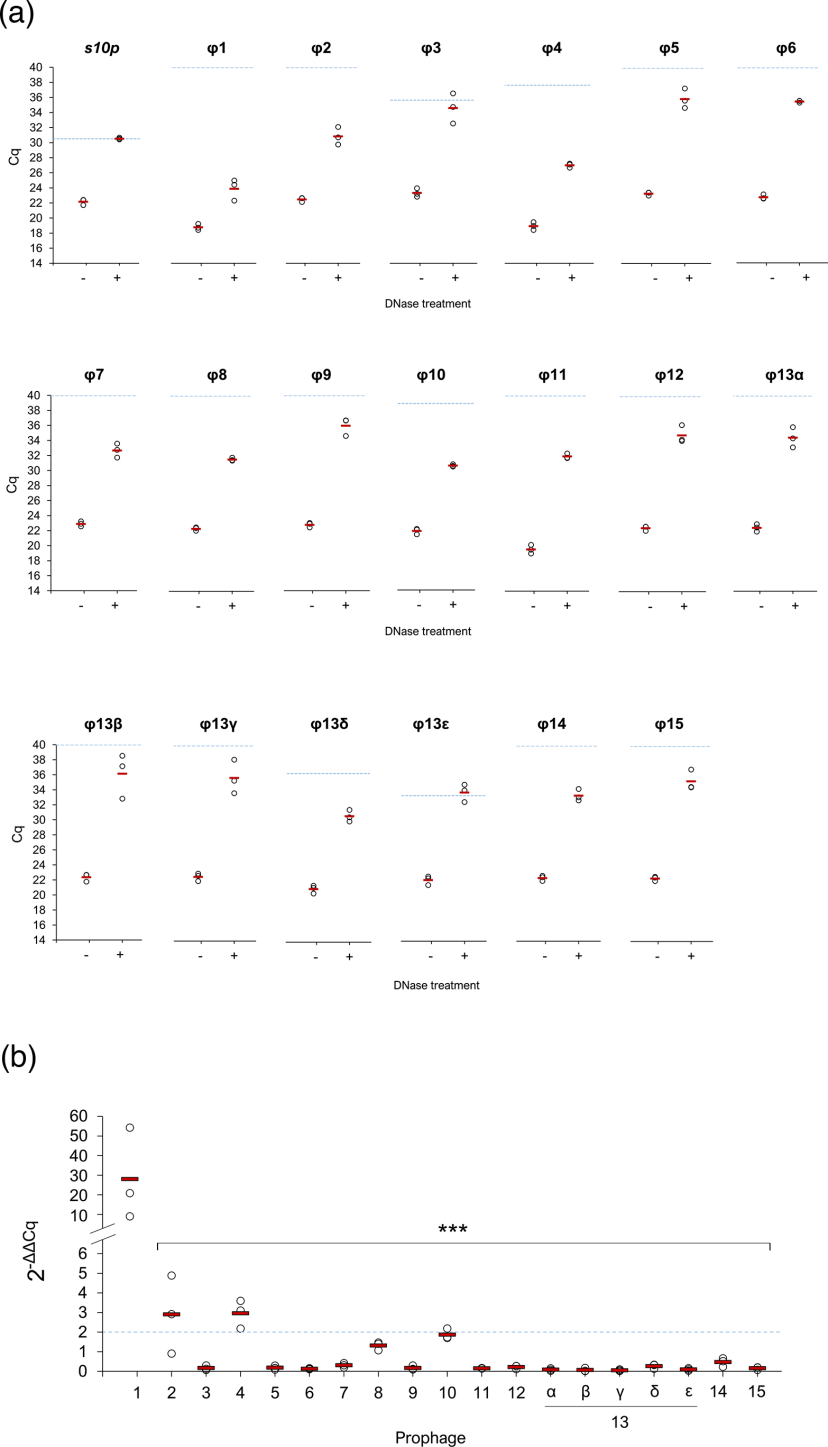
Basal release of *C. clostridioforme* LM41 prophages under non-inducing conditions. *C. clostridioforme* LM41 was diluted 1: 50 from an overnight culture into standard FAB and grown for 24 h under anaerobic conditions. Sterile filtered supernatants were subject to either no treatment or digestion with 10 µg ml^−1^ DNase I for 1.5 h. One microliter of DNase-treated or control supernatant was used as template for qPCR. *C. clostridioforme s10p* (equivalent to *rps*J) was used as the housekeeper and as a marker for the presence of bacterial DNA. (a**)** Raw Cq values for the different target sequences. Data points are from three independent experiments with mean values shown as red bars. NTC Cq values for each target are shown by the dashed blue line. (**b)** Fold change in Cq of phage DNA in supernatant samples following DNase treatment to remove non-encapsidated DNA. The ΔCq values for all target samples were normalized using the ΔCq for the *s10p* gene (DNase treated Cq–untreated Cq), and fold changes were calculated using the 2^-ΔΔCq^ calculation. Data points are from three independent experiments with mean values shown as red bars. The dashed blue line indicates a twofold change threshold for reference. Asterisks denote statistically significant differences between the mean 2^-ΔΔCq^ values for φ1 and the other phages, tested using a repeated measures ANOVA with Tukey post hoc tests (no Greisenham correction), where *P* values ranged <0.0001–0.0002.

Using a dual approach, we examined the profile of phages released following spontaneous induction for our subsequent experiments. Firstly, qPCR was used to identify the presence of encapsidated DNA (indicative of phage) in LM41 culture supernatants. Briefly, filtered supernatants were divided into duplicate samples, of which one was DNase treated and the other was kept as an untreated control. DNase treatment enabled the differentiation of encapsidated phage DNA, which is protected from degradation by the phage protein capsid, from DNA present in the sample that has been released from lysed bacterial cells. For all targets, DNase treatment reduced the quantity of DNA present in the sample (Fig. 4a). Following the treatment, the levels of the bacterial small ribosomal subunit protein 10 (*s10p*) housekeeper gene were reduced below the respective no template control (NTC), suggesting a comprehensive degradation of bacterial DNA in the sample. In contrast, except φ13ε, each of the phage targets remained detectable relative to their respective NTCs, suggesting the presence of phage particles in the supernatant, albeit often at extremely low levels. The differences in mean Cq values between treated and untreated samples were lowest for φ1 (5.11), φ4 (8.06), φ2 (8.37) and φ10 (8.69), suggesting that these phages were most abundant. Indeed, quantification of the levels of each phage in the sample relative to the *s10p* housekeeper was performed using the 2^-ΔΔCt^ method and showed that φ1 was the most abundant phage in the sample (mean±sd: 28.45±23.35), followed by φ4 (2.95±0.72), φ2 (2.90±1.99) and φ10 (1.87±0.26) (Fig. 4b).

**Fig. 5.**
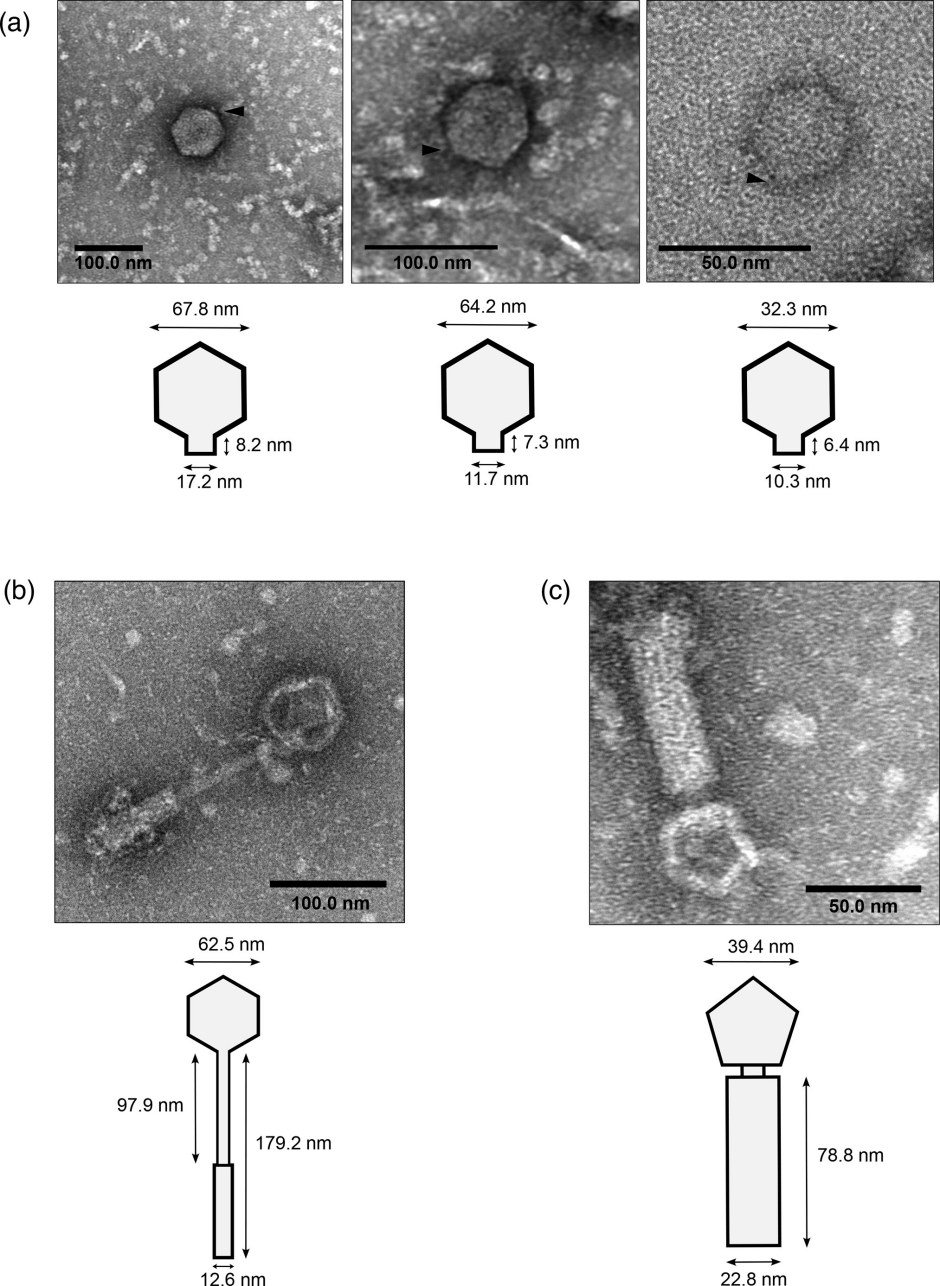
TEM examination of LM41 culture supernatant reveals phage particles. *C. clostridioforme* LM41 was diluted 1:50 from an overnight culture into standard FAB and grown for 24 h under anaerobic conditions. Sterile filtered supernatants were subject to digestion with 10 µg ml^−1^ DNase I for 1.5 h to remove non-encapsidated DNA and then concentrated following 10 % PEG and 1 M NaCl precipitation. Samples were fixed and negatively stained with 0.5 % (w/v) uranyl formate on copper-coated carbon grids and then imaged using a JEOL 1400 Flash TEM running at 80 kV. Particle dimensions for putative podoviruses [(a) *n*=3] and tailed particles [(b, c) *n*=1] displayed in the TEM images are indicated on the schematics. Putative tail projections are indicated by black triangles (A).

To visualize the phage particles spontaneously released into the culture supernatant, we DNase treated filtered culture supernatants of LM41 and then NaCl-PEG 8000 precipitated the phage capsids, which were subsequently imaged using negative staining and TEM. Few particles consistent with phage virions could be identified, most likely because basal release results in extremely low phage titres. However, we were able to identify 14 icosahedral particles, 2 of which had obvious tail-like structures (Fig. 5). The particles lacking tails had diameters in the range 32.4–67.9 nm (mean 54.96±11.59, *n*=12) (examples are shown in Fig. 5a), while the tailed particle in Fig. 5b had a capsid diameter of 62.5 nm, which is consistent with the dimensions of staphylococcal phages with similar genome sizes [36]. We also observed one other tailed particle with a 39.4-nm-diameter capsid (Fig. 5c). Some virion particles appeared to have short protrusions (~7–8-nm length) emanating from the capsid, raising the possibility that they are podoviruses (Fig. 5a). The other structures observed were consistent with siphovirus or myovirus morphology, with the smaller diameter particle associated with what appears to be a large tail structure approximately 78.8 nm in length and 22.8-nm wide (Fig. 5c). Though it is impossible to determine from the imaging analysis which LM41 phages are present in the sample, it is likely that the majority of the podovirus-like particles observed are LM41φ1 given that our previous experiment indicated that φ1 is the most abundant phage and is predicted to have podovirus morphology.

### Accessory genes

We next sought to determine whether there was an obvious advantage to LM41 in maintaining so many prophage sequences. Phages often carry accessory genes that do not directly contribute to the lysogenic lifecycle but that may provide a benefit to the host bacterium by altering its phenotype in a process known as lysogenic conversion [37]. Importantly, accessory genes may also be retained as part of cryptic (defective) prophages [38]. We examined LM41φ1–15 for the presence of accessory genes that may confer some sort of benefit to the LM41 host cell. No products classically associated with bacterial morons (‘more-on’s’), such as exotoxins or immune-evasion factors, were observed in any of the prophages encoded by LM41. This was not necessarily surprising, as *C. clostridioforme* is a member of the healthy gut microbiota and is not usually considered to be virulent. It should, however, be stated that many of the putative ORFs encoded by these prophages are predicted to be hypothetical proteins, so the presence of such factors cannot be definitively ruled out. Similarly, Comprehensive Antibiotic Resistance Database (CARD) analysis indicated that while LM41 carries components of tetracycline and vancomycin resistance gene clusters on its chromosome (Supplementary Material), no antibiotic resistance genes were detected in any prophage region.

We did note the presence of several potentially interesting proteins, which can be broadly grouped as RM system components, diversity-generating elements, hypothetical proteins with some similarity to large polyvalent proteins, phage defence factors, anti-phage defence factors and proteins with possible roles in adaptation within the host intestine (Table 3).

**Table 3.** Prophage-encoded accessory Orfs of interest

Prophage	ORF	Annotation*	PHROG ID	Predicted product	Putative function
1	4	eggNOG-mapper	–	Dextransucrase activity	Host metabolism
	52	PHANOTATE	1048	Anti-CRISPR†	Anti-phage defence
	64	PHANOTATE	–	Hypothetical protein	Unknown
2	4	PHANOTATE	87	IrrE family metalloendopeptidase	Lysogeny regulation
	51	PHANOTATE	1423	Reverse transcriptase	Phage replication, diversity generation
3	13	eggNOG-mapper	2889	PFAM formylglycine-generating sulfatase enzyme	Host metabolism
	25	PHANOTATE	28 876	Metal-dependent phosphohydrolase	Host metabolism
	71	eggNOG-mapper	2889	PFAM formylglycine-generating sulfatase enzyme	Host metabolism
	73	PHANOTATE	3206	Avd protein of DGR	Diversity generation
	76	PHANOTATE	1423	Reverse transcriptase	Phage replication, diversity generation
4	55	PHANOTATE	–	Anti-CRISPR†	Anti-phage defence
	67	PHANOTATE	–	Hypothetical protein	Unknown
5	1	Iterative search	–	Amidoligase enzyme	Host metabolism, phage defence
	4	eggNOG-mapper	16 694	Type I RM system R subunit	Phage defence
	5	eggNOG-mapper	–	Type I RM DNA specificity domain	Phage defence
	8	eggNOG-mapper	–	Type I RM DNA specificity domain	Phage defence
	9	eggNOG-mapper	–	Type I RM DNA specificity domain	Phage defence
	10	eggNOG-mapper	2668	Type I RM DNA specificity domain	Phage defence
	11	eggNOG-mapper	2713	Type I RM system methyltransferase subunit	Phage defence
	12	eggNOG-mapper	19 315	IrrE N-terminal-like domain	Lysogeny regulation
	33,34	PHANOTATE	1384	Mom-like DNA modification protein	Anti-phage defence
	60	eggNOG-mapper	–	Chloramphenicol phosphotransferase-like protein	Resistance against ribosomal peptidyltransferases
	63	eggNOG-mapper	32 044	Toxin SymE, type I toxin–antitoxin (TA) system	Translation repression
6	20	PHANOTATE	1423	Reverse transcriptase	Phage replication, diversity generation
	30	PHANOTATE	424	Phosphoadenosine phosphosulfate reductase	Host metabolism
8	57	PHANOTATE	1423	Reverse transcriptase	Phage replication, diversity generation
	60	PHANOTATE	937	Haemolysin	Host fitness/cell lysis
9	27	PHANOTATE	392	Metal-dependent hydrolase	Host metabolism
	48	PHANOTATE	424	Phosphoadenosine phosphosulfate reductase	Host metabolism
	102	PHANOTATE	16 724	Endonuclease; PHROG indicative of toxin element of TA system	Phage defence
	110	PHANOTATE	1384	Mom-like DNA modification protein	Anti-phage defence
	111	PHANOTATE	10 089	DarB-like antirestriction	Anti-phage defence
	124	Iterative search	1048	Anti-CRISPR†	Anti-phage defence
	149	PHANOTATE	937	Haemolysin	Host fitness/cell lysis
10	2	PHANOTATE	497	TA system HicB-like	Phage defence
	3	PHANOTATE	353	HicA toxin	Phage defence
	49	Iterative search	–	Lar-like restriction alleviation protein	Anti-phage defence
12	4	PHANOTATE	87	IrrE family metalloendopeptidase	Lysogeny regulation
	78	PHANOTATE	1423	Reverse transcriptase	Phage replication, phage defence
13(α)	5	eggNOG-mapper	–	Acetyltransferase	Host metabolism
	28	eggNOG-mapper	32 044	Toxin SymE, type I TA system	Translation repression
	35,37	PHANOTATE	2443	Amidoligase enzyme	Host metabolism, phage defence
	39	PHANOTATE	2520	Gamma-glutamyl cyclotransferase	Host metabolism
	50	eggNOG-mapper	2737	Addiction module antitoxin, RelB DinJ family	Phage defence
	51	eggNOG-mapper	2455	ParE toxin of type II TA system, parDE	Phage defence
13(β)	83	PHANOTATE	310	Transposase	Phage replication
	84	PHANOTATE	296	DNA transposition protein	Phage replication
	95	eggNOG-mapper	8929	Transposase DDE domain	Phage replication
	96	eggNOG-mapper	34 740	PFAM transposase, IS4 family protein	Phage replication
	98	eggNOG-mapper	12 132	Reverse transcriptase (RNA-dependent DNA polymerase)	Phage replication, diversity generation
	104	eggNOG-mapper	34 860	Antitoxin component of a TA module	Anti-phage defence
	105	eggNOG-mapper	–	RM system: HsdM N-terminal domain	Phage defence
	106	eggNOG-mapper	3830	RM system: DNA specificity	Phage defence
	107	eggNOG-mapper	1468	RM system: type III restriction	Phage defence
13(δ)	140	PHANOTATE	310	Mu transposase, C-terminal	Phage replication
	141	PHANOTATE	296	DNA transposition protein	Phage replication
	149	PHANOTATE	424	Phosphoadenosine phosphosulfate reductase	Host metabolism
	155	PHANOTATE	4681	Antitoxin from a TA system	Anti-phage defence
13(ε)	206, 208	PHANOTATE	2443	Amidoligase enzyme	Host metabolism, phage defence
	210	PHANOTATE	2520	Gamma-glutamyl cyclotransferase	Host metabolism
14	40,42	PHANOTATE	424, 2302	Phosphoadenosine phosphosulfate reductase	Host metabolism
	73	PHANOTATE	1423	Reverse transcriptase	Phage replication, diversity generation

1*Annotation was performed using PHANOTATE (Pharokka) or eggNOG-mapper/iterative search with mmseqs against the PHROG database (PhageScope). PHANOTATE annotation calling was given priority for ORF function prediction over PhageScope unless no function could be assigned.

2†Identified as encoding an anti-CRISPR protein by PhageScope using Anti-CRISPRdb.

Proteins with roles in type I RM were identified in LM41φ5, LM41φ13β and LM41φ13ε. Prophage LM41φ5 encodes a complete type I RM system, encompassing three specificity subunits (*hsd*S), a DNA methyltransferase and a restriction subunit. LM41φ13β also encodes a complete type I RM system comprising of HsdM (an S-adenosyl methionine [SAM]-dependent DNA methyltransferase), HsdS (specificity subunit) and HsdR (endonuclease subunit R), where subunits HsdM and HsdR have high homology with similar proteins in *Ruminococcus* sp. (both 97.31 % identity), while HsdS shares some similarity with a protein from *Anaerosporobacter faecicola* (60.22 % identity). Interestingly, we also identified an ORF predicted to have limited similarity (61.11 % identity) to the *E. coli* restriction alleviation protein, Lar (also known as RalR), in LM41φ10. Lar functions to modulate the activity of the *E. coli* K-12 host RM systems in order to protect the Rac prophage from destruction [39].

**Fig. 6.**
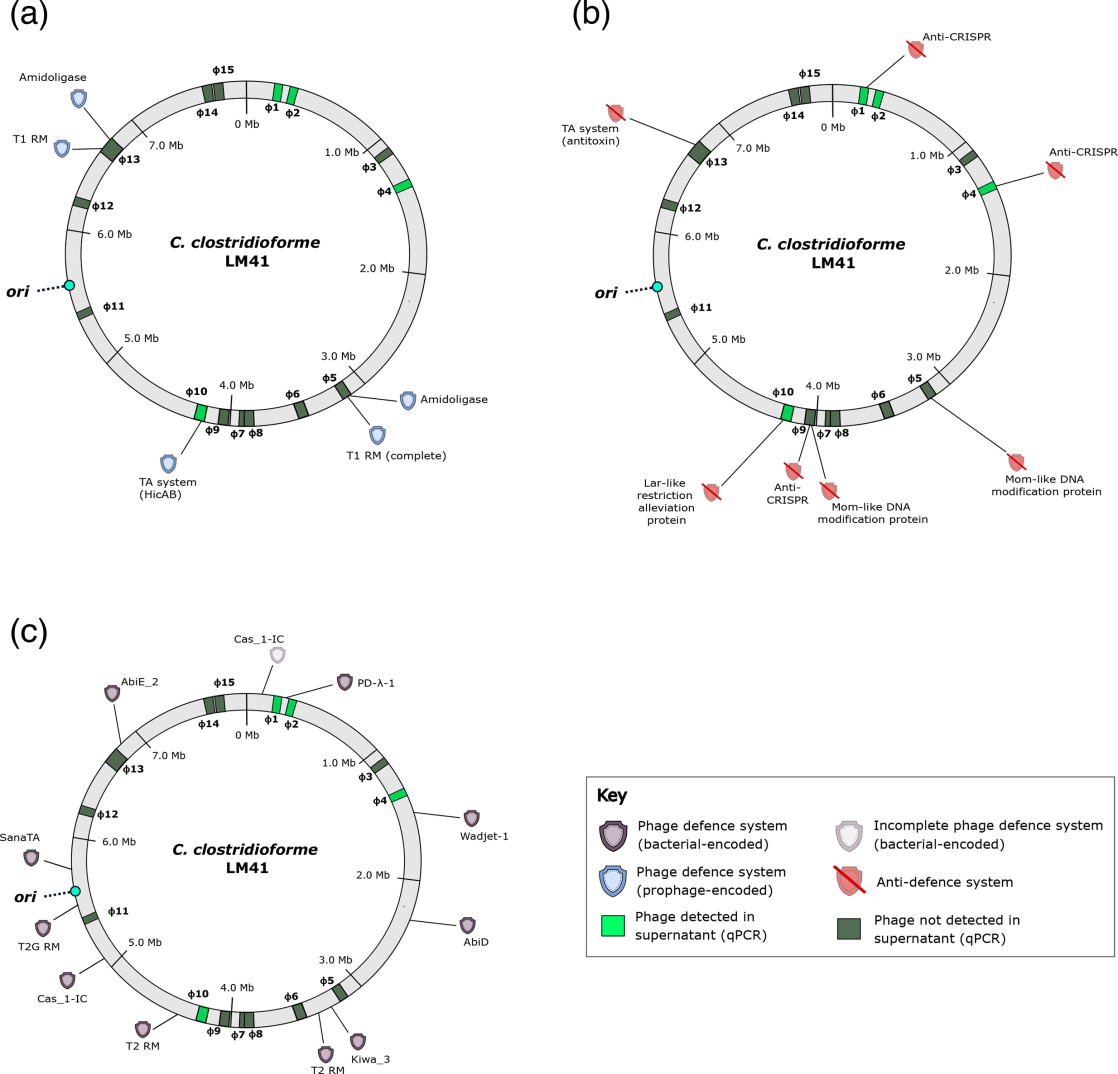
*C. clostridioforme* LM41 and its prophages encode a variety of phage defence and anti-phage defence systems. (a) Defence systems located on prophages. (b**)** Anti-phage defence systems located on prophages. (c**)** Defence systems encoded on the bacterial chromosome. Maps show the bacterial chromosome in grey, with prophage regions highlighted in green. The released phages are denoted in a lighter shade of green. The bacterial chromosome origin of replication (*ori*) is indicated by the turquoise circle. Image generated using Inkscape v.1.

In addition to the RM systems, we also noted the presence of a number of other factors associated with potential phage defence and anti-phage defence systems (Fig. 6). Multiple different toxin–antitoxin (TA) systems were associated with the LM41 prophages: a HicB/HicA-type system identified in LM41φ10, a RelB/RelE-type system in LM41φ13α and a SymE-like type I toxin in both LM41φ5 and LM41φ13α. Amidoligase enzymes were also identified in prophages φ5 and φ13α. In terms of anti-phage defence systems, anti-CRISPR systems were identified in φ1, φ4 and φ9, while a predicted TA system antitoxin was observed in φ13δ.

The presence of diversity-generating elements was also noted in multiple LM41 prophages. Group II intron reverse transcriptase/maturase proteins were identified in prophages LM41φ2 (Orf51), LM41φ6 (Orf20) and LM41φ12 (Orf78), while LM41φ3 is predicted to carry both a reverse transcriptase/maturase family protein (Orf76) and a homologue of *Bordetella* phage BPP-1 diversity-generating retroelement protein Avd (Orf73), which in BPP-1 facilitates sequence variation in target protein genes, enabling changes in host cell surface factors [40,41].

Proteins with potential roles in influencing adaptation of the bacterial lysogen within the host intestinal environment were also observed. These include Orf60 of LM41φ8 and Orf149 of LM41φ9, which encode putative haemolysins, and factors influencing host metabolism, such as gamma-glutamyl cyclotransferases in φ13α and φ13ε, an ORF with dextransucrase activity in φ1 and phosphoadenosine phosphosulfate reductases in φ13δ and φ14.

Finally, prophages LM41φ1 and LM41φ4 encode an unusually large (8 kb) ORF at their 3′ ends, which constitutes almost 20 % of the phage genome sequence. Both ORFs are predicted to be hypothetical proteins but have some limited similarity (53.84 % ID) to large polyvalent protein-associated domain 3 from *Podoviridae* sp., suggesting that they could play a role in protecting and establishing the phage DNA when it enters a new host cell [42].

## Discussion

*C. clostridioforme* LM41 has an atypically large genome for this species, with a strikingly high proportion of DNA attributed to MGEs [18]. This work sought to characterize the prophage sequences associated with this strain to determine whether they might contribute to its enhanced fitness in the dysbiotic gut. The interrogation of the LM41 genome sequence revealed poly-lysogeny: 15 prophage-derived sequences – comprising >9.6 % of the bacterium’s 7.78 Mb genome – were observed, many with genomic organization and size reminiscent of the well-characterized Gram-positive staphylococcal siphovirus phages [43]. Four of the LM41 phages are predicted to encode all of the necessary modules for functionality, with a further nine phages requiring additional characterization. We attempted to test the functionality of the LM41 prophages experimentally using chemical induction; however, the classical SOS-inducing antibiotics mitomycin C, norfloxacin and ciprofloxacin failed to induce bacterial lysis or significantly increase the quantity of encapsidated DNA released from LM41 compared to the untreated control, suggesting that LM41 prophages do not respond efficiently to this type of inducing signal. This observation is not necessarily surprising as work in *Clostridioides difficile* has shown that some prophages respond more effectively to fluoroquinolone antibiotic exposure than to the ‘gold standard’ mitomycin C [44]. Furthermore, others have shown that in a variety of species of human gut bacteria, fewer than one-quarter of prophages predicted to be functional using bioinformatics could be induced under experimental conditions [45]. This may suggest higher than expected rates of cryptic phage carriage in these bacteria or could mean that prophages in these species have different inducing signals to those from classically studied hosts such as *E. coli* and *S. aureus*. Arguably, it is likely that other prophage-inducing signals occur in the gut environment given the lack of potent DNA-damaging agents typically present in physiological habitats, and recent work has shown that in *Vibrio* spp., prophage-encoded transcription factors can activate small proteins which induce their prophage in an SOS-independent manner [46], while *S. aureus* prophage phiMBL3 can be induced independently of the SOS response by a pyocyanin metabolite from *Pseudomonas aeruginosa* [47]. Accordingly, further work is necessary to screen a variety of inducing agents against LM41 prophages before they can be conclusively determined to be functional or defective.

Within lysogenic populations, spontaneous prophage induction can occur in a small proportion of cells, leading to the release of low titres of phage into the surrounding environment [48]. Molecular examination of LM41 culture supernatants confirmed that LM41φ1, φ4 and φ10 particles are spontaneously released, supporting our prediction of these prophages as functional. φ2 was also detected, suggesting that this phage is functional despite scoring <100 for completeness. TEM imaging showed that spontaneously produced particles are predominantly podoviruses, though the observation of other putative phage particles with longer tails indicates diversity in the morphological characteristics of LM41 phages. We also observed diversity among the lysogeny control systems utilized by the different prophages, suggesting the existence of a diverse community of phages within the *Lachnospiraceae* that can employ different mechanisms to maintain their latent state within their host bacterium.

Three regions were also observed that contained phage remnants to varying degrees, with the defects present predicted to abolish the ability of these phages to excise, replicate and/or package efficiently. LM41φ13 was revealed to be not just one phage, but a 136-kb region of phage remnants, presumably derived from excessive or uncontrolled recombination events. A similar Mu-type phage is present in the *C. clostridioforme* LM41 relative *Lachnoclostridium* sp. YL32 (accession: CP015399), where two copies of the 35.8 kb phage sequence are arranged divergently at genome locations 3 363 626–3 399 467 bp and 3 591 511–3 627 351 bp, with each of these sequences displaying high similarity (95.82 % ID, 89 % cover, *E* value 0.0) to the δ region of LM41φ13, suggesting the potential for a common ancestor. It is unclear as to how and why the LM41φ13 region became so variable in LM41. In contrast to many well-characterized lysogenic phages, transposable phages do not excise out of the chromosome in order to proliferate [49]. In the case of the archetypal phage Mu, the integrated phage replicates by looping the bacterial chromosome and cleaving the DNA, enabling the formation of Shapiro intermediate structures whereby the prophage sequence is duplicated and integrated into new sites on the bacterial chromosome at random, in a mechanism similar to a transposon [49]. We can see no obvious explanation for the hypervariability observed in region LM41φ13; however, given the apparent propensity for DNA acquisition by strain LM41, it is possible that this strain has lost some of the mechanisms required for maintaining fidelity in DNA recombination and repair, resulting in the loss of intact phage regions. Additional work will be required to evaluate this theory further.

Given the quantity of prophage DNA carried by LM41, we hypothesized that one or more of the resident prophages contributes to the fitness of the host bacteria in the dysbiotic intestine. The examination of the prophage sequences for the presence of morons (accessory genes with functions not linked to lysogeny) revealed no obvious candidates for the enhanced fitness displayed by LM41 in the gut environment. We did, however, find that the LM41 prophages carry a number of potentially interesting genes, including those with roles in phage defence and anti-phage defence. Defence systems include a variety of DNA methyltransferases, a number of specificity subunits and two complete type I RM systems for the modification of DNA, presumably to aid phage defence against degradation by the host bacterium’s RM systems. Plasmid carriage of orphan HsdS (specificity) subunits that can interact with chromosomally encoded HsdM (methylation) and HsdR (restriction) subunits has been described in *Lactococcus lactis*, creating a molecular expansion pack for the host cell type I RM repertoire without requiring carriage of a full HsdMSR system [50]. It is tempting to speculate that a similar combinational system utilizing phage-encoded specificity or methyltransferase subunits with native restriction and/or methylation components may contribute to the ability of LM41 to accept foreign DNA if it can be recognized and methylated by these enzymes prior to the destruction by the host cell’s restriction systems, possibly lending some explanation as to why this strain appears to have gained so much horizontally acquired DNA compared to its most closely related strains. Further to this, we observed a protein in LM41φ10 with limited similarity to the *E. coli* restriction alleviation protein, Lar, which has a role in modulating the activity of *E. coli*-encoded RM systems to protect prophage DNA [39]. It is not impossible that the Lar-like protein of LM41φ10 exerts global impacts upon its host organism and that this could function synergistically with the other phage-encoded RM components to retain foreign DNA in LM41. In order to test this theory, a phage-cured strain of LM41 would need to be generated and its ability to accept exogenous DNA compared with the parental LM41 and with variants carrying defined combinations of prophages. Unfortunately, given the paucity of genetic tools to permit manipulation of this organism, such experiments are not currently possible.

Other putative defence systems include TA systems, which can facilitate phage defence at the population level, inducing processes such as abortive infection following infection of the host cell or by inhibiting virion formation [51,52]. We also observed factors with roles in modifying the host cell surface to prevent superinfection of lysogens, potentially acting similarly to the amidoligase of *E. coli* phage phiEco32, which modifies cell wall receptors to prevent adsorption by competing phages [53]. As bacteria and their phages are engaged in a constant arm race, the evolution of anti-phage defence systems on the part of the phage is necessary to overcome bacterial defences. DefenseFinder [54] analysis of the LM41 genome revealed a variety of phage defence systems carried on the bacterial chromosome, including Wadjet-1, AbiD, AbiE, Kiwa-3 and a type I-C CRISPR-Cas system (Fig. 6c), that may interfere with phage reproduction. Interestingly, LM41 φ1 and 4 encode anti-CRISPR systems, and no regions of homology to either phage could be detected in the spacer sequences from the bacterial CRISPR array. Other LM41 phages carry solitary TA system antitoxin components or restriction alleviation proteins with potential roles in subverting phage defence systems. It is currently unclear whether these antitoxins are part of degenerate TA systems or whether these proteins could act as anti-phage defence systems by enabling the phages to counter toxins from other host- or phage-encoded TA systems.

Group II reverse transcriptase/maturase proteins were also detected in a number of prophages. The role of these proteins for phage or host cell biology is unclear. Indeed, it is possible that these elements have been acquired elsewhere and have become integrated within the prophage sequences, as seems likely in the case of LM41φ6 where the putative helicase ORF has been interrupted by the insertion of a putative group II reverse transcriptase/maturase protein. This hypothesis is further supported by the fact that 29 LtrA group II intron sequences are present throughout the LM41 genome, suggesting that these are a feature of the host rather than the phages.

Finally, we detected two putative haemolysin proteins carried by prophages φ8 and φ9. It is possible that these are misannotations, as φ9 Orf149 shows high homology to CHAP domain-containing protein from *Enterocloster* sp. (BLASTp: 99 % query cover, *E* value 0.0, 98.45 % ID to accession WP_256170368.1), which is predicted to have a role in peptidoglycan hydrolysis, suggesting a role in phage-mediated cell lysis. However, if these proteins are indeed haemolysins, they could potentially provide LM41 with an advantage in the gut, perhaps by scavenging iron from the host via haemolysis. The ability of LM41 to lyse erythrocytes could be evaluated *in vitro* to examine whether LM41 has the potential to participate in nutrient acquisition in this way.

Phages are the most numerous entities within the gut microbiome [55], and yet, the phages associated with the members of the microbiota remain poorly characterized. Here, we have identified an interesting example of poly-lysogeny in *C. clostridioforme* strain LM41 and have utilized bioinformatic tools and experimental approaches to offer insight into some of the characteristics of these phages, shedding light on their potential impact upon their host bacterium.

## Abbreviations

AAI: amino acid identity
CARD: Comprehensive Antibiotic Resistance Database
CFX: ciprofloxacin
EDTA: ethylenediaminetetraacetic acid
FAB: Fastidious Anaerobe Broth
HTH: helix-turn-helix
ICE: integrative conjugative element
MC: mitomycin C
MGE: mobile genetic element
NCBI: National Center for Biotechnology Information
NFX: norfloxacin
NTC: no template control
qPCR: quantitative PCR
RM: restriction–modification
TA: toxin–antitoxin
TEM: transmission electron microscopy
VLP: virus-like particle
